# When doctors falter: Resilience in modern obstetrics and gynecology, a narrative review

**DOI:** 10.1002/ijgo.71327

**Published:** 2026-10-01

**Authors:** Elizabeth Tunney, Daniel Kane, Mike Geary

**Affiliations:** ^1^ Department of Obstetrics and Gynaecology Royal College of Surgeons in Ireland, Rotunda Hospital Dublin Ireland; ^2^ Rotunda Hospital Dublin Ireland

**Keywords:** burnout, patient safety, physician grit, physician resilience, psychological wellbeing, stress, workplace challenges

## Abstract

The practice of obstetrics and gynecology (O&G) is frequently regarded as one of the most demanding medical specialties, with physicians being called to duty at any hour of the day or night, with acute patient demands requiring prompt management. Consequently, high levels of physician burnout and psychological distress have been reported among O&G healthcare professionals, with these challenges further compounded by the COVID‐19 pandemic. Despite the prevalence of mental health difficulties, many healthcare professionals do not seek out professional intervention and support. Within O&G, trainees appear vulnerable, with studies reporting significant rates of stress, anxiety, depression, and burnout. These mental health challenges contribute to reduced job satisfaction, increased intentions to leave specialist training programs, and growing concerns regarding physician attrition and workforce retention. In this context, physician resilience (i.e., the ability to excel in the face of adversity) emerges as an area worthy of exploration. This narrative review explores the multifaceted emotional and practical elements of physician resilience and grit in contemporary obstetrics and gynecology practice. A comprehensive literature search was conducted using PubMed and the Royal College of Surgeons in Ireland library database, employing terms related to physician wellbeing, burnout and interventions specifically related to obstetrics and gynecology. Inclusion criteria were limited to full text, English‐language articles published within the past 10 years to ensure relevance to contemporary practice. The review examines the interrelationship between physician burnout, mental health challenges among O&G trainees, and their impact on attrition rates. We aim to highlight the need for practical interventions at institutional levels with consideration of environmental/workplace factors, which might improve physician wellbeing and grit, strengthen workforce retention, and ultimately optimize patient care.

## 1 INTRODUCTION

Obstetrics and gynecology is a high‐stress medical field with substantial burnout rates reported internationally.^1^ Burnout and psychological distress among physicians are increasingly recognized as threats to physician wellbeing, psychological safety, patient safety and healthcare workforce sustainability.^2^ Occupational stress occurs among physicians when the perception of the demands of a situation exceed the resources available to the individual physician to meet these demands.^3^ In O&G, these demands arise from a combination of environmental and individual factors. Contributory organizational factors, such as inadequate staffing, excessive workloads, long working hours, workplace culture, and insufficient renumeration for work completed, have all been associated with physician distress, while contributory personal factors, including self‐efficacy, coping style, and a propensity toward avoidant behaviors, may enhance individual vulnerability to workplace stress.^3^ There is evidence to suggest that burnout and chronic workplace stress could increase the incidence of clinical errors, compromise patient safety, and might be associated with increased patient mortality.^4^ The consequences of physician distress extend beyond workplace performance. The medical profession continues to face intense scrutiny regarding physician mental health and suicide, an issue that historically has been difficult to address openly within the profession itself.^5^ Physicians have been identified as one of several occupational groups at an elevated risk of suicide.^6^ It is estimated that one physician dies by suicide every day in the USA and one doctor every 3 weeks in the UK.^7^ The reality of these findings have naturally intensified societal interest in factors that can protect against psychological distress, reduce burnout, and support retention of physicians in specialist training programs.

Physician resilience has emerged as an essential protective factor against the adverse effects of workplace stress and burnout.^8^ It has been defined as the ability of an individual to respond adaptatively to stress and adversity while achieving goals with minimal psychological and physical cost and maintaining mental wellbeing.^9^ Closely related to resilience is the concept of physician grit, which encompasses perseverance and sustained commitment in the pursuit of long‐term professional goals despite adversity.^10^ Physician resilience and grit have been suggested as important attributes that can combat the individual effects of workplace stress, enhance professional fulfillment, and promote workforce retention.^2^, ^9^ However, it is important to note the distinction: modern concepts of resilience suggest it should not be viewed solely as an individual responsibility. Increasingly, the ability of a physician to be resilient is considered a “workplace” issue, whereby workplace resilience depends upon organizational supports and systemic interventions that address modifiable environmental stressors, including staffing levels, workload, workplace culture, and remuneration.^3^ The importance of physician resilience and mental wellbeing became prevalent during the COVID‐19 pandemic, which placed unprecedented demands on healthcare workers, especially those working in critical care and emergency settings.^11^ High rates of stress, anxiety, and depression were reported among frontline healthcare professionals caring for patients with COVID‐19, emphasizing the interaction between challenging work environments and physician psychological safety and wellbeing.^12^

Within O&G specifically, generalized concerns regarding burnout, mental health, and workforce retention have become particularly acute. High levels of burnout and psychological distress among O&G trainees have been described internationally, with studies reporting significant rates of anxiety, depression, and emotional exhaustion.^13^ These challenges contribute to attrition from O&G training programs, with substantial numbers of trainees reporting intentions to leave the specialty or discontinuing formal medical training completely.^14^ Attrition, of this kind, has substantial implications for future workforce projections, including workforce planning, service delivery and the future sustainability of the specialty.^15^

Despite growing recognition of these challenges, there remains limited commentary, either in the formal or gray literature, examining the evolution of physician resilience and grit within contemporary O&G practice. The aim of this narrative review is to explore the concept of physician resilience and grit in O&G, focusing on the multi‐faceted challenges facing O&G physicians. This review examines physician burnout, psychological distress, and mental health issues among O&G trainees, as well as the relationship between these factors and trainee attrition. This paper will also highlight the potentialrole of physician resilience and grit in combination with organizational interventions in supporting physician wellbeing. Crucially, it concludes by reflecting on the need for institutional and systemic‐level approaches to ensure that responsibility for resilience does not rest solely on the individual physician.

## 2 MATERIALS AND METHODS

For this narrative review, two researchers conducted a comprehensive review of the peer‐reviewed and gray literature in September 2025. An electronic search of the PubMed database and Royal College of Surgeons in Ireland library database using the following search terms: “physician resilience” (83 results), “physician burnout” (205 results), “attrition rates” (83 results), “physician grit” (2 results), with “obstetrics,” and “gynecology” added (Appendix S1). We selected papers published between January 2015 and July 2025. The criteria for inclusion in the study was limited to peer‐reviewed articles that provided information on the following: physician burnout in O&G, mental health of O&G trainees, attrition rates in O&G, interventions both personal and organizational to combat burnout/build resilience in O&G, and, finally, institutional and patient care benefits from building psychological safety among physicians.

The reference lists of the included studies were also reviewed. We excluded articles focused on medical students or undergraduate teaching, and endeavored, where possible, to exclude articles examining resilience in professions other than obstetrics and gynecology. Articles not in English were also excluded. A total of 89 articles were included to review modern day resilience among O&G physicians. Titles and abstracts were reviewed by two independent authors, ET & DK, who extracted information regarding study characteristics (if any), central themes relating to burnout/physician resilience, and a full manuscript review of the findings from each paper included. No ethical approval was necessary for this manuscript as it is derived from publicly available literature and did not involve any human participants or identifiable data.

## 3 DISCUSSION

### 3.1 Physician resilience and grit in obstetrics and gynecology

Given the alarming rates of psychological distress and suicide reported among medical physicians worldwide, the concept of physician resilience has emerged as a complex and promising concept. It is acknowledged that O&G physicians face unique challenges as a result of the physical, emotional, and intellectual demands of the specialty and as a group are therefore known to suffer high rates of burnout. Enhancing physician resilience has been targeted as an important part of the path forward away from the deleterious effects of burnout.^16^ Resilience and job satisfaction are often presented in combination as potential resources available to individuals to ameliorate the effects of workplace stress.

Resilience, derived from the Latin term “resilio” to leap or spring back, can be further defined as a “stress coping ability” or “the personal qualities that enable one to thrive in the face of adversity.”^17^ Physicians with resilience demonstrate a set of behaviors and traits that allow an individual to manage difficult work hours, sleep deprivation, clinical working environments, time pressures, the personal challenges of working with sick and/or difficult patients, in addition to feelings of isolation.^18^ Furthermore, personal habits such as a positive attitude, good social supports and physical activity are described as elements that contribute to resilience.^19^ A recent Australian study established the importance of environment and culture when considering physician resilience. They found resilience to be a developmental phenomenon that can be learned, and, while affected by individual attributes as previously detailed, can be strongly influenced by surrounding culture.^20^ Winkel et al. depict resilience as fueled by an individual's dedication to reaching professional goals; however, ultimate attainment of these goals does appear to “require support from the surrounding culture to develop.” Descriptions of personal involvement and the individual physician association with resilience‐building are frequently described in the literature.^21^ However, the alternative view to resilience‐building among the healthcare environment warns against creating an inappropriate interplay between these two concepts. Card et al. distinguish between unavoidable occupational suffering, largely inherent in the physician's role, and avoidable occupational suffering (e.g., healthcare systems failures that can be prevented).^22^ They emphasize that the implementation of resilience training based on the individual as an “antidote” to inherent organizational pathologies is inappropriate and might lead to avoidable suffering and even compound the harm that physicians experience.^22^

In addition to resilience, grit among O&G trainees has been associated with success in medical training and repeatedly examined and reported in the literature.^23^ Grit was conceptualized much earlier than burnout and is an important and relevant concept in the field of O&G. Grit was first described by a team of psychologists from the University of Pennsylvania who theorized that grit was an important determinant associated with career success. Grit, distinct from resilience, is not only about being resilient to combat the failure or adversity associated with a career in medicine but also having persistent and deep commitment toward a particular career goal for a long period of time.^24^ Being gritty therefore entails being resilient and beyond. Often, interventions intended to build resilience among physicians target a lack of supposed “grit” within the individual, which justifies the promotion of personal coping strategies, such as mindfulness and cognitive behavior therapy. The implementation of personal development/coping strategies has been heavily criticized. Chakrabarti et al.^25^ illustrate that often the “fault” is conveyed as lying with the worker when the considerations of resilience‐building are discussed, therefore absolving the healthcare organization of its responsibilities. Often the individualization of blame negates the poor working conditions and unreasonable expectations placed on physicians, who ultimately carry responsibility for the harm. The con of individual resilience,^26^ some argue, is part of the existential problem.

Changing how we speak about resilience shifts the focus to identifying and mitigating the sources of conflict rather than considering how physicians should respond.^27^ We are reminded that resilience is not a dirty word.^18^ Research shows that organization‐focused interventions are more effective, particularly those including multiple elements such as structural changes and promotion of good teamwork and communication.^28^

### 3.2 Physician burnout in obstetrics and gynecology

Physician burnout has been recognized for decades as a significant occupational phenomenon, first defined by Freudenberg in 1974 as “to fail, wear out, or become exhausted by making excessive demands on energy, strength or resources.” Freudenberg described burnout as a “concept with multiple manifestations on the physicians it affected including physical and behavioural signs.”^29^ Burnout was later conceptualized by Maslach et al., who developed the first validated burnout inventory tool and contributed further to the definition of burnout, describing it as a psychological syndrome comprised of “a state of exhaustion in which one is cynical about the value of one's occupation and doubtful of one's capacity to perform.”^30^ This conception has informed contemporary understanding of burnout as a complex condition that affects both physicians as individuals and the healthcare institutions in which they work.

There is consistent evidence demonstrating that physicians who work in obstetrics and gynecology disproportionately experience higher levels of burnout compared to other medical and surgical specialties. Studies have shown that 43% of O&G trainees and 31% of O&G attending physicians experience burnout, compared with 21–24% of doctors (including trainees and attending physicians) in other surgical specialties.^31^, ^32^ These findings suggest that burnout might not be solely attributable to the acknowledged general pressures of medical practice but might be associated with characteristics unique to O&G. Some authors suggest the high prevalence of burnout among O&G physicians might stem from the high‐acuity and rapid turnover of patients, characteristic of O&G practice,^33^ while others have highlighted poor working conditions relating to staff shortages, increased administrative workloads and a perceived lack of work–life balance.^34^ Further, burnout has been linked to a combination of a lack of control over work, lack of regular feedback, and lack of professional development.^35^ Collectively, these findings indicate that burnout arises from the interaction between demanding clinical environments and modifiable workplace conditions, rather than individual vulnerability alone.

The COVID‐19 global pandemic represented an unprecedented healthcare challenge to the world and further highlighted the vulnerability of the O&G workforce to burnout.^36^ O&G doctors found themselves redeployed, with training pathways disrupted, and the pressure of maintaining provision of specialist services during a global health crisis introduced additional stressors.^13^ However, evidence from the UK,^31^ Spain,^37^ and the USA^38^ has demonstrated that approximately one in two O&G doctors experienced burnout before the global pandemic, confirming that physician burnout is a longstanding and systemic problem. The COVID‐19 global pandemic simply exposed underlying deficiencies in healthcare systems and exacerbated pre‐existing workplace pressures for O&G physicians rather than creating burnout itself.^13^

Importantly, the implications of physician burnout extend well beyond the individual physician. High levels of burnout in obstetricians and gynecologists have been associated with increased practice of defensive medicine, poor doctor wellbeing, reduced job satisfaction, and greater potential of workforce attrition.^31^ These associations suggest that the prevalence of burnout might negatively affect clinical decision‐making, professional performance at work, and the future sustainability of the O&G workforce. Given the central role of O&G physicians in the provision of gynecological and maternity care, persistent burnout in the physicians providing this care could affect patient outcomes, service provision, and the quality of the healthcare provided more widely. Therefore, physician burnout must be viewed as a patient safety and workforce sustainability issue rather than an occupational health concern affecting individual physicians.

Despite the evolving body of evidence worldwide, burnout among specialist and trainee obstetricians and gynecologists remains underrecognized, with the lack of published data regarding O&G physicians especially concerning.^4^ The differences in burnout levels visible across specialties further suggests that interventions should be focused on the unique patterns exhibited by each specialty in the target population and be informed by the unique organizational and clinical demands faced by that target population using a multidimensional approach.^39^ Consistent with Maslach et al., awareness that a problem exists can be the first step in alleviating any form of job burnout.^40^ Physicians, policymakers, healthcare leaders, and professional bodies in O&G therefore must prioritize physician burnout as a critical workforce issue. Arguably, efforts to address physician burnout must be afforded similar levels of importance and consultation as initiatives aimed at reducing maternal morbidity and perinatal mortality. O&G policymakers and stakeholders require cognizance of the interconnected relationship between physician wellbeing, workforce retention and the provision of safe, high‐quality patient‐centered care.

### 3.3 Mental health and distress in obstetrics and gynecology

Mental health distress is increasingly recognized as a significant challenge among O&G physicians, with substantial implications for individual wellbeing, workforce retention and patient care. Compared to the general population, female physicians are more likely to die by suicide.^6^ Doctors are overrepresented in suicide statistics, with female physicians particularly vulnerable; their suicide risk has been estimated at 2.27 times the general population, while male doctors' suicide risk slightly is lower at 1.41 times the general population.^41^ Following the COVID pandemic, a nationwide UK survey reported that 62% of doctors experienced anxiety, 31% reported depression, and 9% described suicidal thoughts.^13^ Similar findings were described by the Royal College of Obstetricians and Gynecologists in a survey, which reported 25% of O&G trainees had experienced mental health problems such as anxiety and depression in the previous 12 months.^1^

The consequences of mental health distress among O&G physicians extends beyond manifestations within an individual physician. Poor psychological wellbeing has been associated with reduced job satisfaction, increased absence from work secondary to illness, and workforce attrition; it threatens patient safety and overall satisfaction.^39^ Considering the sustained issues with workforce pressures within O&G, developing an understanding of and addressing physician wellbeing is important. The European Working Directive (EWTD) introduced in 2003 provided limitations on the working hours of physicians, stipulated a new maximum quota of 48 h per week including overtime, and heralded a new era of more resident sleep and shorter shifts.^42^ Analysis of the contemporary literature illustrates that this generation of doctors continue to experience significant levels of depression, anxiety, and suicide compared to the general population.

Despite the increasing prevalence of mental distress among O&G physicians, stigma surrounding mental illness among physicians in medicine remains pervasive.^43^ This stigma can operate at both societal and provider levels^44^ and can be even more extreme in low‐income countries.^45^ A survey exploring senior doctors' beliefs and attitudes regarding mental health issues within the medical profession found evidence of changing perspectives across generations, with a greater appreciation of individual wellbeing and its value demonstrated within younger physician cohorts. Nevertheless, a general suspicion toward mental illness prevailed, with marked reluctance to report individual mental illness to a fellow medical colleague noted.^46^ Historically, physicians have been characterized as occupying a superior space, where they consider themselves “special and above the mental illnesses that might grip their patients.”^47^ This perception is at odds with the role of the modern physician in O&G, who integrates in a medical community where mental illness exists within general public health at large.^6^ While such attitudes might be diminishing, stigma persists and continues to pose a significant barrier to physicians seeking support and might contribute to psychological distress and reduced resilience among individual physicians.

In a recent report reviewing mental health and the wellbeing of doctors, the General Medical Council (GMC) outlined the ABC of doctors' core needs, which, when met, might help to ensure physician wellbeing and motivation at work. These core needs include autonomy/control, belonging, and competence.^48^ However, further longitudinal research is required to evaluate the long‐term efficacy and impact of implementing these interventions when trying to improve physician wellbeing. While large volumes of qualitative research have explored mental distress and the wellbeing of physicians in the immediate period following the COVID‐19 pandemic, there remains a scarcity of data specifically focusing on resilience and mental health among O&G residents in the current period following the COVID‐19 pandemic.^37^ Exploration of these factors is vital, given their implications for physician wellbeing, workforce sustainability, staff retention, patient satisfaction, and patient safety.

Resilience has emerged as an important construct in the understanding of how physicians in O&G respond to occupational stress and psychological adversity.^49^ However, it is acknowledged that resilience does not eliminate exposure to workplace stressors entirely.^50^ Higher levels of resilience might mitigate the effects associated with mental health distress and support adaptation to changing clinical environments.^51^ Resilience, therefore, might act as a protective factor against mental health distress and positively influence psychological wellbeing.

### 3.4 Attrition in obstetrics and gynecology training

Attrition is neither new nor unique to the medical profession and constitutes a significant decrease in workforce numbers.^25^ O&G experiences some of the highest rates of attrition among medical specialties, with 12% of attending physicians leaving the specialty within 3 years of attaining their certificate of training, with an estimated 30% of trainees exiting the specialty during training.^22^ Compared with other specialties, including general surgery (20%), vascular surgery (15%), and emergency medicine, attrition rates in O&G remain disproportionately high, emphasizing the necessity to understand the factors that influence trainee retention and the resilience that might retain them.

In relation to attrition in O&G, physician resilience has been highlighted as a key factor when considering physician wellbeing and career sustainability.^52^ However, the concept of resilience as an individual issue rather than an organizational responsibility remains problematic. High‐quality O&G training environments that incorporate supportive supervision, constructive feedback, professional respect, and opportunities for career development might increase job satisfaction, reduce attrition, and promote excellence in education and training.^53^ Conversely, it has been reported that trainees working within poor, unsupportive learning environments, particularly those exposed to bullying, undermining behaviors, and inadequate supervision, might perform suboptimally at work and have a diminished sense of belonging, which might contribute to a higher degree of attrition.^53^

Institutional factors contribute significantly when considering the experiences of trainees engaged in specialist O&G training. Chronic pressures of rota gaps, stretched resources, increasing service demands and ever‐increasing patient numbers place significant pressure on O&G physicians, including those attendings who have been in the profession the longest.^52^ These working conditions might result in an excessive workload, obstruction to training opportunities, and persistent physician fatigue, which can, over time, erode resilience and contribute to professional dissatisfaction and diminished wellbeing. Sleep deprivation, burnout, and clinician health are intricately intertwined and must be recognized as contributors toward trainees considering leaving the specialty.^54^

The number of UK graduates opting for careers in O&G remains low,^55^ which is curious given that medical students tend to have a positive experience during their undergraduate exposure to O&G.^55^ This suggests that factors encountered during postgraduate training might have more significant influence on recruitment and retention than exposure while at medical school. Nevertheless, many contributors have argued that contemporary healthcare systems characterized by increasing managerialism intensify workforce issues and contribute to issues with job satisfaction and staff retention.^56^ Further, this perceived shift in power from clinicians to managers in addition to a change in emphasis from patient care commitments to primary concerns with budgetary efficiency have reduced professional autonomy and diverted attention away from patient‐centered care.^57^

The complexity of the organizational issues outlined might explain why interventions such as the EWTD, which reduced working hours, have not eradicated the problem of attrition and peri‐attrition among O&G trainees.^14^ Consequently, efforts to improve retention should move beyond individual‐focused solutions and address the organizational conditions that influence resilience. Solution‐based approaches that involve creating enhanced quality of learning environments, reducing excessive workloads, addressing sleep deprivation and improving job satisfaction might represent important strategies for improving job satisfaction, strengthening resilience and reducing attrition rates among O&G trainees.

### 3.5 Strengths and limitations

To our knowledge, this is one of few reviews that focuses specifically on physician resilience and grit as it relates to the modern practice of O&G. The strengths of this review include the inclusion of diverse commentaries, including research derivatives from high‐income academic institutions and output from low‐income and resource‐limited research centers, thus reflecting the global prevalence of issues related to physician wellbeing in obstetrics and gynecology. The inclusion of narrative commentaries in addition to survey studies enhances the practical relevance of this article, as an attempt to connect the multifaceted elements that make up physician resilience. The authors concede that this narrative review does not include a systematic search or recognized prescribed quality appraisal protocols; therefore, the risk of a selection bias cannot be ruled out. There is a paucity of literature regarding O&G resilience and O&G physicians specifically. Therefore multiple studies/reviews are drawn from general literature specific to healthcare providers in general. Considering the sparse nature of commentary regarding physician resilience in relation to O&G specifically, this review reveals key contributory themes. The identified themes hold the potential to illustrate the foundations of resilience‐building among clinical educators, program directors and other important stakeholders involved in developing the structure of postgraduate medical training.

## 4 CONCLUSION

Resilience among O&G physicians is a concept that extends beyond the individual physician and must be considered within a broader organizational and healthcare system context. The use of the term “burnout” in O&G has drawn criticism secondary to the conveyance of singular responsibility resting solely on the distressed physician, thereby concealing the multifaceted structural and institutional challenges that contribute significantly to physician distress. Considering the high levels of burnout and exhaustion reported among O&G physicians in the literature, urgent attention is required from healthcare institutions, policy‐makers, and other relevant stakeholders. Contemporary research should prioritize the development and evaluation of organizational interventions that promote physical and psychological safety in the workplace, which could complement individual supports for physicians. Further investigation should focus on comprehensive system‐level measures that reinforce the nuanced understanding of physician resilience described in this paper, as the product of a combination of personal and organizational factors. Such approaches have the potential to enhance physician wellbeing and resilience and by consequence improve patient safety and reduce clinical risk through the creation of increasingly functional healthcare environments.

## AUTHOR CONTRIBUTIONS

**Elizabeth Tunney:** Conceptualization, methodology, investigation, writing – original draft preparation. **Dan Kane:** Conceptualization, writing – review and editing. **Mike Geary**: Conceptualization, writing – review and editing. All authors agree with the final version of the manuscript and its submission to the journal.

## FUNDING INFORMATION

This study was not funded.

## CONFLICT OF INTEREST STATEMENT

The authors declare no conflict of interest.

## Supporting information


Appendix S1.

## Data Availability

Research data are not shared.
