# Supplementary material for: When doctors falter: Resilience in modern obstetrics and gynecology, a narrative review
Source: Int J Gynaecol Obstet. 2026 Oct 1;175(1):86–92. doi: 10.1002/ijgo.71327 (PMC13629582; doi:10.1002/ijgo.71327)
Supplement: Supplementary file 1 [file IJGO-175-86-s001.docx]

Data S1

Structure of the Search Strategy in **PubMed**

Supplemental Material S1 presents the keywords used to search the literature across one major medical literature database – PubMed – on 22^nd^ September 2025.

TABLE S1. Search terms used for database

| **Search terms for literature review** | **Search terms for specialty specific** |
| --- | --- |
| “physician burnout*”, “physician resilience*”, “attrition rates*”, “physician grit*” | obstetrics*, gynecology* |

Our search strategy combined the search terms within each column from Supplemental Material S1 the Boolean operator “OR”. All search terms from column 1 were then combined with those in column 2 using the Boolean operator “AND”. Database filters were used to restrict search results to papers published between January 2015 to July 2025, excluding papers with outdated research quality.
